# Occurrence of Ochratoxin A in Grape Juice of Iran

**Published:** 2018

**Authors:** Jamal Yusefi, Mehran Valaee, Firouzeh Nazari, Javad Maleki, Ehsan Mottaghianpour, Roya Khosrokhavar, Mir-Jamal Hosseini

**Affiliations:** a *Department of Food Safety and Hygiene, School of Public Health, Zanjan University of Medical Sciences, Zanjan, Iran*; b *Food and Drug department of Iran University of Medical Sciences, Tehran, Iran.*; c *Food and Drug Laboratory Research center, Ministry of Health and Medical Education, Tehran, Iran. *; d *Department of Pharmacology and Toxicology, School of Pharmacy, Zanjan University of Medical Sciences, Zanjan, Iran. *; e *Zanjan Applied Pharmacology Research Center, Zanjan University of Medical sciences, Zanjan, Iran.*; 1 J.Y and M.V. Contributed equally to this work.

**Keywords:** Ochratoxin A (OTA), Grape juice, High-performance liquid chromatography (HPLC), Maximum Residue level (MRL), Iran

## Abstract

Ochratoxin A (OTA) is one of the most important mycotoxins that contaminate a broad range of agricultural and food products. In this study, the occurrence of OTA in available brands of grape juice in Iran purchased from retail outlets or producer were determined for the first time using high-performance liquid chromatography (HPLC) with immunoaffinity columns(IAC) as the clean-up step. The average recoveries for OTA in grape juice ranged from 54.2 to 86.6% with the coefficient of variation lower than 17.3% in lowest spiked level (0.5 µg/L). The estimated LOD and LOQ of OTA were 0.04 µg/L and 0.125 µg/L, respectively. In our study, 70 samples of grape juice evaluated for OTA content. The results showed that in 39 out of 70 samples (55.7%) OTA levels were above the LOQ with the maximum level of 2.6 µg/L and the mean contamination was 0.5 µg/L. Although the mean contamination of OTA in analyzing samples was lower than the MRL set by EU, the high incidence of contamination in these products is worried. Considering the importance of OTA in public health, control of pre- and post-harvest, storage and grape juice manufacturing process, such as HACCP, GAP, and GMP recommended preventive measures are required.

## Introduction

Mycotoxins have a significant impact on human and animal health via effects on organelles such as kidney, liver, and immune system and also induction of teratogenic and carcinogenic effects. They caused giving rise in economic problems due to their effect on health and rendering commodities unacceptable for national or international trade (1-4). Ample evidence has propounded production of OTA as one of the important mycotoxins by Aspergillus and Penicillium genius (5-8). Based on the previous studies, it was suggested that Balkan endemic nephropathy, endemic fatal renal disease, in certain rural areas of Bulgaria, Romania, and Yugoslavia is related to OTA exposure (9). In this respect, the International Agency for Research on Cancer (IARC), has classified OTA as a carcinogen of the group 2B, possible human carcinogenic compound (10). There are some evidences for OTA being implicated in esophageal cancer in north-eastern of Iran (11).

Emerging lines of research suggested that OTA has been found in various raw and processed food commodities all over the world, including cereals, coffee, beer, cocoa, nuts, beans, peas, bread, and rice (3, 5, 13-17). Many investigations suggested that the presence of OTA in dried fruits, grapes and by grain products such as grape juice and wine is the result of the contamination of grape surface, commonly occurring before and after the harvest period (16). Therefore, the Joint FAO/WHO Expert Committee on Food Additives (JECFA) has established the provisional maximum weekly intake (PMWI) of 120 ng OTA/kg body weight (17). 

In this context, a growing body of evidence indicates the detection of OTA in pork and poultry meat, blood, kidneys, and milk of pig as well as in human blood and mother’s milk (18, 19-20).

Also, some evidences have reported the OTA contamination in grape juice in different countries such as Switzerland (21), Germany (22), France (23), Canada (24) and Turkey (25). It proposed temperature, rain, and relative humidity that are the main and favorable factors in OTA production in grapes (21-25). The results of Zimmerli and Dick (1995) in red grape juice showed the OTA contamination in 8 samples in the range of 3-311 ng/kg (21). Other study in German investigated grape juice samples purchased between 1995 and 1998 and found that 81.6% of white grape juice and 89% of red grape juice samples contained OTA (26). Another work in Germany showed 78 percent of 37 white and red grape juice samples were contaminated with OTA (22). Furthermore, 54%, 12.9%, and 20% of collected grape samples in southern France, Canada, and Turkey were contaminated by OTA, respectively (23-25). 

The natural occurrence of OTA in grape juice sold in Iran is only limited to Ghafari *et al. *(27) and Gholampour Azizi (28) using ELISA methods with 1.2 and 35% contamination in analyzed grape juices, respectively.

Since OTA has been reported in various studies as a grape, wine, and grape juice contaminant, EU set a maximum level of 2 µg/kg OTA in wine and grape juice (29). In this context, some evidences are accumulating to show that HPLC associated with fluorescence detection has become the most popular method for OTA detection and quantification in wine and grape beverages (22- 25, 30-32). Therefore, the objective of this study was to determine the concentration of OTA in the red grape juice sold in Iranian supermarkets in 2014-2015 for the first time using with immunoaffinity columns (IAC) and HPLC method coupled with fluorescence detection.

## Experimental

*Chemicals and reagents*

Methanol and acetonitrile (both LC gradient grade), disodium hydrogen phosphate, Sodium chloride, potassium dihydrogen phosphate, potassium chloride, and hydrochloric acid were purchased from Merck (Darmstardt, Germany). The standard of OTA was obtained from Sigma-Aldrich (Sigma: St Louis, Mo, USA). The Ochratoxin A IAC was purchased from Libios (Puri-Fast OTA IAC, libios, France). Polyethylene glycol (PEG) 8000 was purchased from Aldrich (Sigma: St Louis, Mo, USA).

*Sample Collection*: The analysis was limited only to the red grape juices produced in 2014-2015. A total of 70 samples of red grape juices were analyzed. Sixty domestic samples from variant brands and batches and 10 imported samples from South Korea and Germany were collected from the retail markets in Iran**.** Samples were kept in 4 °C until analysis.

*Apparatus*

Detection and quantification were performed using a HPLC system (KNAUER, Berlin, Germany) equipped with an isocratic pump Model 1000 (KNAUER, Berlin, Germany) and fluorescence detector (KNAUER, Berlin, Germany). Instrument control and data acquisition were performed with a personal computer running the open LAB chromatography data systems (CDC) EZchrom Elite (KNAUER, Berlin, Germany). The wavelengths setting used were at 333 and 477 nm as excitation and emission wavelength, respectively, in OTA determination. The analytical column was an RP-C18 (250 ×4.6 mm i.d, particle size 5 μm, KNAUER, Berlin, Germany).

**Table 1:** Results of validation assessment of HPLC method developed for determination of OTA in grape juice (n = 3

**Spike level** **(**µg/L**)**	**Recovery%**	**RSD%**
0.5	54.2	17.3
1.0	70.4	6.4
2.5	77.8	3.6
5.0	86.6	2.8

**Table 2:** Performance criteria for Ochratoxin A (EU, No 519/2014).

**Level µg/L**	**Ochratoxin A**		
	**RSD**_r_ **%**	**RSD**_R_ **%**	**Recovery %**
< 1	40≥	≥ 60	50 to 120
≥ 1	20≥	≥ 30	70 to 110

**Table 3:** Contamination data for OTA in grape juice samples marketed in, Iran.

**70**	**NO. of samples**
39(55.7)	Sample positive (%)
0.52	Mean^a^ (µg/L)
2.69	Max (µg/L)

a : Mean of positive samples

**Table 4:** Natural occurrence of OTA in grape juice samples marketed in Iran

**NO. of samples(%)**	**Range**
(44.3)31	< LOQ
(54.3) 38	LOQ ≥OTA ≥ MRL
1(1.4)	> MRL

LOQ = 0.125 µg/L

MRL: 2 µg/L

**Figure.1:**
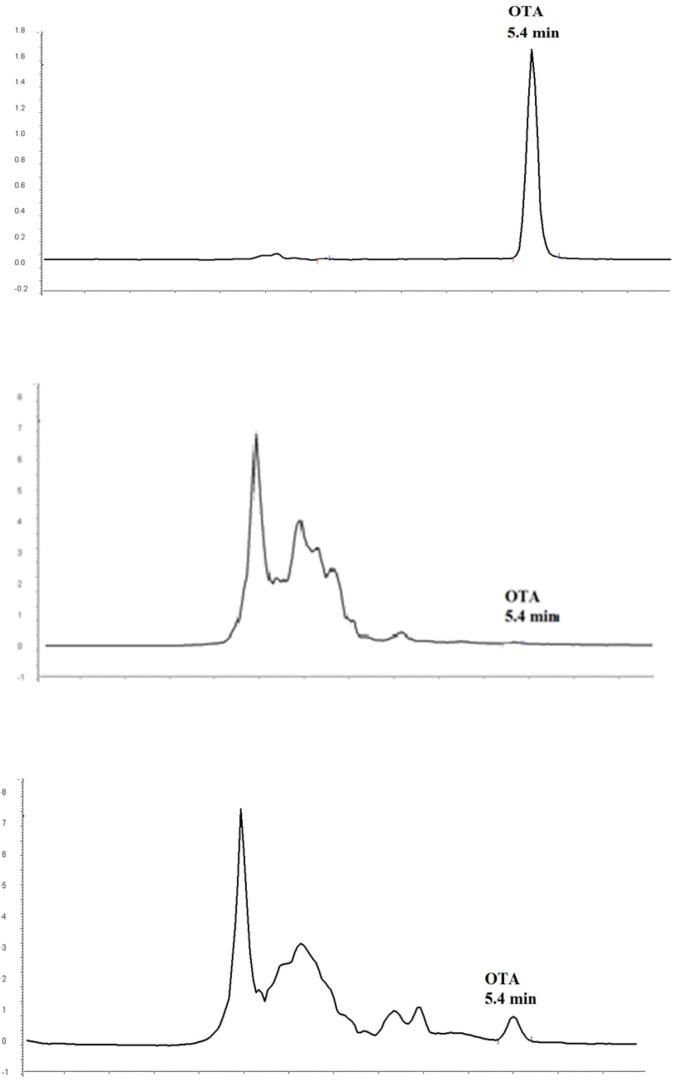
Chromatograms of OTA standard at 1µg/L (A) blank grape juice sample (B) naturally contaminated grape juice sample with OTA at 0.26 µg/L (c

*Standard, sample and solvent preparation*

The OTA stock standard solution was prepared at the concentration of 200 µg/L and stored at –18 °C in a mixture of toluene: acetic acid, 99:1 (v/v). In order to prepare the OTA standard solution, suitable volume of the stock solution was evaporated using a gentle stream of nitrogen and dissolution of the residue in the mobile phase. 

The standard 1µg/mL was used in preparation of a series of OTA calibration curve solution (0.125, 0.25, 0.5, 1, 2 and 5µg/L) using mobile phase. The working standard solutions were stored at 4 °C.

Phosphate buffered saline (PBS) solution used in the IAC cleanup procedure was prepared as follows: 8.0 g of sodium chloride, 1.2 g of disodium hydrogen phosphate, 0.2 g of potassium dihydrogen phosphate and 0.2 g potassium chloride were dissolved in approximately 990 mL water and the pH value was adjusted to 7.4 with concentrated hydrochloric acid. The solution was made up to the volume of 1000 mL with deionized water.

A solution of 15% sodium chloride and 2% sodium bicarbonate was prepared in distilled water. The dilution solution was contained Polyethylene glycol (1%) and NaHCO_3_ (5%) in distilled water.

*Procedure*

OTA was quantiﬁed according to the method of EU 14133/2003 with minor modification (33). Briefly, 35 mL of red grape juice diluted (1:1, v/v) with dilution solution and shake vigorously for 3 min to mix. Then, the IAC was preconditioned (10 mL PBS) and the mixture of grape juice and dilution solution passed from the IAC with slight gravity effect or vacuum. The IAC was washed with 10 mL of deionized water to elimination of interfering substances and dried with air. The retained OTA was eluted with 2 mL of pure methanol and mixed with 3mL deionized water. This final solution was vortex (1min) and 100 µL were injected into the HPLC.

*Chromatographic Condition*

The separation was performed on a C_18_ analytical column using an HPLC system equipped with fluorescence detector. The mobile phase including glacial acetic acid–water–acetonitrile (2:99:99, v/v/v) was filtered through a 0.45 mm membrane before use. The flow rate was 1 mL/min^-1^. The fluorescence detector was operated at anexcitation wavelength of 365 nm and emission wavelength of 477 nm. 

*Method validation*

To evaluate the reliability of the results, in addition to evaluate linearity, limits of detection (LOD), limit of quantification (LOQ), accuracy, and precision of method were evaluated by performing recovery. In each working day, a blank and a spiked sample were also analyzed. The samples were spiked with OTA concentrations at level of 0.5,1, 2.5, and 5 µg/L. The validation process consisted of assessing the following parameters, precision, and recovery.

## Results and Discussion

OTA contamination was estimated in collecting red grape juice samples using immunoaffinity column and HPLC method coupled with fluorescence detection. The method was validated in terms of linearity, LOD and LOQ, precision and recovery. The method was satisfactory in terms of selectivity as an immunoaffinity column that was applied for purification of OTA, which eliminated false positive results caused by interfering materials. A typical chromatogram obtained from a naturally contaminated red grape juice sample with 0.26 µg/L OTA is shown in Figure 1. Linearity was assessed for OTA over a range of 0.125-5 µg/L and the correlation coefficient was greater than 0.999. The estimated LOD (signal to noise ratio = 3) and LOQ (signal to noise ratio = 9-10) of OTA were 0.04 µg/L and 0.125 µg/L, respectively.

Accuracy of the method was assessed by performing recovery experiments and the results were correct based on the recovery values. Each test was performed and quantified in triplicate. Also, the suggested method was applied in the testing of 60 grape juice samples from Iran and 10 samples from foreign countries. The average recoveries and relative standard deviations (RSD) were in the range of 54-87 µg/L and 3-8%, respectively (Table 1), which are in accordance with EC 401/2006 (34) (Table 2).

Results from the survey of commercial grape juice for OTA contamination are presented in Table.3. Although, no positive results of OTA presence were obtained in the imported grape juices; the data showed that the OTA level in 31 out of 70 samples was lower than the LOQ 0.125)µg/L) and OTA contamination amount was between 0.14 and 2.68 µg/L in 39 samples (Table 3 and Table 4).

As previously mentioned, there are little data on OTA contamination in the grape juice in Iran using the ELIZA kit method. Ghafari *et al*. (27) analyzed a total of 85 red and white grape juices, and reported OTA detected only one red grape juice sample (1.2%) at the level of 1.6 ng/mL. The results of Gholampour Azizi study (28) showed that 32% of samples containing OTA (from 100 samples) were higher than 10 mg/kg OTA. The levels of OTA were in the range of 2.1-18.4 µg/L with the mean concentration 8.14 µg/L. This result showed the mean and the maximum level of OTA is higher than our results due to applied method. Although, ELISA method is a favorable, rapid and simple approach in low volume samples which needs a minimal cleanup procedures, the antibodies used in this method often show cross-reactivity to compounds similar to mycotoxins (35). The result of OTA contamination in 39 wine samples in Slovakia using HPLC technique showed that 50% of the samples contained detectable amounts of OTA in the range of 0.01-0.46 ppb which is lower than EU standards (2 µg/L36).

Also, investigations in southern France showed that 54% of collected grape samples were contaminated by OTA (23). Miraglia and Brera (15) reported that OTA levels in German grape juice samples were higher than 5.3 µg/L.The results of investigations in Germany (83.3%), UK (95%), Norway (96.4%), Italy/France (72.7%), and Spain (100%) are in agreement of our data and showed the highest incidence of OTA in grape juice. The results of analyzing samples in Canada, USA, and Chile showed lower incidence (12.9 and 2.9%) of OTA in grape juice samples (5, 24).

## Conclusion

Although in our samples, only one sample was contaminated with more than EC regulatory limits for OTA (2 µg/L), the highest incidence of contamination (55.7%) was observed in analyzing samples. Due to the fact that children are the main consumers of juice and juice drink products, further and routine surveys of OTA contamination is required in order to control and reduce the intake of OTA. Furthermore, to determine the possible health risks of the consumption of grape juice in Iran, it needs to calculate the daily intake and consumption of grape juice. Considering the importance of OTA in public health especially in children, control of pre- and post-harvest, storage and grape juice in the manufacturing process, such as HACCP, GAP and GMP, as well as preventive managements which are highly required was recommended.

## References

[1] Larcher R, Nicolini G (2001). Survey of ochratoxin A in musts, concentrated musts and wines produced or marketed in Trentino (Italy). J. Commodity Sci.

[2] Yazdanpanah H (2006). Mycotoxin Contamination of Foodstuffs and Feedstuffs in Iran. Iran. J. Pharm. Res.

[3] Yazdanpanah H (2011). Mycotoxins: Analytical Challenges. Iran. J. Pharm. Res.

[4] Mottaghianpour E, Nazari F, Mehrasbi MR, Hosseini M-J (2017). Occurrence of aflatoxin B1 in baby foods marketed in Iran. J. Sci. Food Agric.

[5] Vega M, Ríos G, Baer DV, Mardones C, Tessini C, Herlitz E, Saelzer R, Ruiz MA (2012). Ochratoxin A occurrence in wines produced in Chile. Food Control.

[6] Lasram S, Oueslati S, Mliki A, Ghorbel A, Phillipe S, Chebil C (2012). Ochratoxin A and ochratoxigenic black Aspergillus species in Tunisian grapes cultivated in different geographic areas. Food Control.

[7] Varga J, Kevei E, Rinzu E, Teren J, Kozakiewicz Z (1996). Ochratoxin production by Aspergillus species. Appl. Environ. Microbiol.

[8] Rosa C, Magnoli CE, Fraga ME, Dalcero AM, Santana DMN (2004). Occurrence of ochratoxin A in wine and grape juice marketed in Rio de Janeiro, Brazil. Food Addit. Contam.

[9] Battaglia R, Hatzold T, Kroes R (1996). Occurrence and significance of ochratoxin in food. Food Addit. Contam.

[10] World Health Organization International Agency for Research on Cancer (2002). IARC Monographs on the Evaluation of Carcinogenic Risks to Humans. Volume 82 Some traditional herbal medicines, some mycotoxins, naphthalene and styrene.

[11] Lacey J (1998). The microbiology of cereal grains from areas of Iran with a high incidence of esophageal cancer. J. Stored Prod. Res.

[12] Quintela SM, Villarán C, López de Armentia I, Elejalde E (2013). Ochratoxin A removal in wine: A review. Food Control.

[13] Nazari F, Sulyok M, Yazdanpanah H, Kobarfard F, Krska R (2014). A survey of mycotoxins in domestic rice in Iran by liquid chromatography tandem mass spectrometry. Toxicol. Mech. Method.

[14] Yazdanpanah H, Miraglia M, Calfapietra FP, Brera C (2001). Natural occurrence of aflatoxins and ochratoxin A in corn and barley from Mazandaran and Golestan provinces in north of I. R. Iran. Mycotoxin Res.

[15] Battilani P, Pietri A (2002). Ochratoxin A in grapes and wine. Eur. J. Plant. Pathol.

[16] Mule G, Susca A, Logrieco A, Stea G, Visconti A (2006). Development of a quantitative real-time PCR assay for the detection of Aspergillus carbonarius in grapes. Int. J. Food Microbiol.

[17] Ochratoxin A, JECFA (2000). Joint FAO/WHO expert Committee on Food Additives. Safety Evaluation of Certain Food Additives and Contaminants. WHO/FAO Food Additives Series 44. IPCS-International Programme on Chemical Safety.

[18] Kuiper-Goodman T, Scott PM (1989). Risk assessment of the mycotoxin ochratoxin A. Biomed. Environ. Sci.

[19] Höhler D (1998). Ochratoxin A in food and feed: occurrence, legislation and mode of action. Zeitschrift für Ernährungswissenschaft. J. Nutr. Sci.

[20] Skaug MA (1999). Analysis of Norwegian milk and infant formulas for ochratoxin A. Food Addit. Contam.

[21] Zimmerli B, Dick R (1995). Determination of ochratoxin A at the ppt level in human blood serum, milk and some foodstuffs by high-performance liquid chromatography with enhanced fluorescence detection and immunoaffinity column clean-up: methodology and Swiss data. J. Chromatogr B. Analyt. Technol. Biomed. Life Sci.

[22] Woese K (2000). Ochratoxin A in grape juice and wine. Mycotoxin Res.

[23] Sage L, Kribovok S, Delbos E, Seigle-Murandi F, Creppy EE (2002). Fungal flora and ochratoxin A production in grapes and musts from France. J. Agric. Food Chem.

[24] Ng W, Mankotia M, Pantazopoulos P, Neil RJ, Scott PM (2004). Ochratoxin A in wine and grape juice sold in Canada. Food Addit. Contam.

[25] Akdeniz AS, Ozden S, Alpertunga B (2013). Ochratoxin A in dried grapes and grape-derived products in Turkey. Food Addit. Contam. Part B Surveill.

[26] Wolff J (2000). Ochratoxin A in cereals and cereal products. Arch. Lebensmittelhyg.

[27] Ghafari Z, Kazemi Ghahfarokhi N, Rahimi E (2011). Presence of Ochratoxin A in red and white grape juice Commercialized in Iran. Am-Euras. J. Toxicol. Sci.

[28] Gholampour Azizi E (2012). Evaluation of the presence of ochratoxin in grape juices and raisins. J. Food Technol. Nutr.

[29] EC (2006). Commission Regulation No. 1881/2006. Off. J. EU.

[30] Belli N, Marin S, Sanchis V, Ramos AJ (2002). Ochratoxin A (OTA) in Wines, Musts and Grape Juices: Occurrence, Regulations and Methods of Analysis. Food Sci. Technol. Int.

[31] Aboul-Enein HY, Kutluk OB, Altiokka G, Tunel M (2002). A modified HPLC method for the determination of ochratoxin A by fluorescence detection. Biomed. Chrom.

[32] Terra MF, Prado G, Pereira GE, Ematné HJ, Batista LR (2013). Detection of ochratoxin A in tropical wine and grape juice from Brazil. J. Trop. Agric. Food Sci.

[33] European Committee for Standardization (CEN) (2003). Foodstuffs-Determination of ochratoxin A in wine and beer-HPLC method with clean-up on a immunoaffinity column.

[34] EC (2006). Commission regulation No. 401/2006. Off. J. EU.

[35] Zheng MZ, Richard JL, Binder J (2006). A review of rapid methods for the analysis of mycotoxins. Mycopathologia.

[36] Belajove E, Rauova D (2007). Determination of ochratoxin A and its occurrence in wines of Slovakian retail. J. Food Nutr. Res.

